# Chronic kidney disease increases cardiovascular unfavourable outcomes in outpatients with heart failure

**DOI:** 10.1186/1471-2369-10-31

**Published:** 2009-10-21

**Authors:** Arise GS Galil, Hélady S Pinheiro, Alfredo Chaoubah, Darcília MN Costa, Marcus G Bastos

**Affiliations:** 1 NIEPEN - Interdisciplinary Nucleus of Study and Research in Nephrology, Federal University of Juiz de Fora; Center for Control of Hypertension, Diabetes and Obesity, Juiz de Fora, Brasil

## Abstract

**Background:**

Chronic heart failure (CHF) has a high morbidity and mortality. Chronic kidney disease (CKD) has consistently been found to be an independent risk factor for unfavorable cardiovascular (CV) outcomes. Early intervention on CKD reduces the progression of CHF, hospitalizations and mortality, yet there are very few studies about CKD as a risk factor in the early stages of CHF. The aims of our study were to assess the prevalence and the prognostic importance of CKD in patients with systolic CHF stages B and C.

**Methods:**

This is a prospective cohort study, dealing with prognostic markers for CV endpoints in patients with systolic CHF (ejection fraction ≤ 45%).

**Results:**

CKD was defined as estimated glomerular filtration rate <60 mL/min/1.73 m^2 ^and CV endpoints as death or hospitalization due to CHF, in 12 months follow-up. Eighty three patients were studied, the mean age was 62.7 ± 12 years, and 56.6% were female. CKD was diagnosed in 49.4% of the patients, 33% of patients with CHF stage B and 67% in the stage C. Cardiovascular endpoints were observed in 26.5% of the patients. When the sample was stratified into stages B and C of CHF, the occurrence of CKD was associated with 100% and 64.7%, respectively, of unfavorable CV outcomes. After adjustments for all other prognostic factors at baseline, it was observed that the diagnosis of CKD increased in 3.6 times the possibility of CV outcomes (CI 95% 1.04-12.67, p = 0.04), whereas higher ejection fraction (R = 0.925, IC 95% 0.862-0.942, p = 0.03) and serum sodium (R = 0.807, IC 95% 0.862-0.992, p = 0.03) were protective.

**Conclusion:**

In this cohort of patients with CHF stages B and C, CKD was prevalent and independently associated with increased risk of hospitalization and death secondary to cardiac decompensation, especially in asymptomatic patients.

## Background

Chronic heart failure (CHF) is considered a public health problem, in view of its high costs and increasing number of hospital admissions [1-4]. In Brazil, the public health system, *Sistema Único de Saúde *(SUS), is responsible for more than 75% of the hospitalization for CHF, which generates a large social and financial burden [5]. Despite implementation in recent years of effective strategies to reduce the mortality of patients with CHF, the mortality rates resulting from the disease still remain high, and are directly related to the duration and frequency of hospitalization [1,2,5-7]. Similar to the presence of symptoms in heart failure, the presence of hospitalization secondary to CHF decompensation, *per se*, predicts cardiovascular mortality [7-9].

Chronic kidney disease (CKD) is associated with a high risk of cardiovascular events [10-12]. It is an independent risk factor for adverse outcomes, recurrence of hospitalization, and in comparison with the general population, increases by 15 to 30 times cardiovascular (CV) mortality [12,13]. In fact, death mainly due to CV causes is far more common than dialysis at all stages of CKD [14].

The prevalence of CHF among CKD patients is elevated and equally predicts mortality [13-16]. CHF is a common cause of renal impairment, and its decompensation is a major cause of CKD progression [15]. On the other hand, reduced glomerular filtration (GF) linearly associates with increased prevalence of CHF [15,17].

Most studies which assess the negative impact of CKD and other factors in the curse of CHF have been done in patients with most severe form of the disease (NYHA Class IV of CHF), usually in a hospital setting [4,8,14-16,18]. Recently, CHF patients with asymptomatic systolic dysfunction were evaluated, and the presence of CKD was associated with a greater mortality [17].

Despite the incentives, the prognostic classification of CHF has rarely been applied in practice. This may be one of the explanations for the scarcity of studies evaluating risk factors and adverse outcomes in outpatients with CHF [1-3]. In the present study, we aimed to investigate the prevalence of CKD and its possible association with cardiovascular outcomes in patients with systolic CHF stage B and C.

## Methods

### Subjects

Eighty-three patients treated at the Center for Control of Hypertension, Diabetes and Obesity - SCHDO, in the city of Juiz de Fora- MG, were observed from the period of January through December of 2006.

SCHDO is a municipal unit of secondary outpatient clinic that offers free treatment to hypertensive patients in stage 3, group C, complications of type 2 diabetics, type 1 diabetics with difficult clinical control, 3^rd ^degree obese patients with associated morbidities, and those referred from other municipal basic health care units.

This was an observational study during which the patients were prospectively followed for 12 months, for surveillance of cardiovascular outcomes. Data were collected at the study baseline and after 12 months. All patients confirmed participation by signing the informed consent form, and the study was approved by the Ethics Committee of the Federal University of Juiz de Fora.

The patients were selected among those with CHF who were attended bimonthly at the SCHDO. The patients were taking medications that optimize CHF treatment, including angiotensin-converting enzyme inhibitors and/or angiotensin receptor blocker, beta-blocker (carvedilol), furosemide, and digital when indicated. Patients over the age of 18 years, regularly attending the Clinic in the last six months, with CHF stages B and C, and ejection fraction by bi-dimensional Doppler echocardiogram of less than or equal to 45% were included [2]. Patients with active infectious-contagious diseases, neoplasia, and systolic CHF possibly reverted, such as valvular or coronary lesions with indication for percutaneous or surgical correction, were excluded from the study.

The patients were classified according to the chronological staging of CHF, in stages B and C. Stage B was characterized when there was a structural heart disease and no symptoms (NYHA class I symptoms), and stage C when the structural disease was associated with previous or current symptoms (NYHA class II or III symptoms) [1,3]. Demographic data, such as age and sex, were evaluated, as well as cardiovascular risk factors. Obesity was diagnosed when the body mass index (BMI) was greater than 30 kg/m^2^; sedentariness was considered when the regular physical activity was less than 30 minutes per day and less than 3 times per week; smokers were identified as those who smoke at least one cigarette per day for a period of more than six months; arterial hypertension was considered when blood pressure levels were above 140 × 90 mmHg, or when on antihypertensive treatment; diabetes mellitus was diagnoses when the fasting blood glucose levels were over 100 mg/dL, or when using an oral hypoglycemic medication or insulin [19-22].

The diagnosis of ischemic cardiopathy (IC) was based on previous history and/or documented upon hospitalization by angina, acute myocardial infarction, myocardial revascularization, percutaneous transcoronary angioplasty, or coronary angiography with injuries consistent with coronary artery disease [23]. Pulse pressure (PP) was considered abnormal when values were equal or higher than 53 mmHg [24]. Laboratory parameters and respective cut-offs for normality (in brackets) were: serum hemoglobin (>12 g/dL), fasting blood glucose (<100 mg/dL), total cholesterol (<200 mg/dL), LDL-cholesterol (<100 mg/dL), triglycerides (<150 mg/dL), and serum sodium (140 a 148 mEq/L) [20,25].

The echocardiographic parameters studied were left ventricular ejection fraction (including those patients with percentage ≤ 45%), measures of left atrium diameter, and of left ventricular diastolic diameter, due to their importance as prognostic parameters in CHF [1,3].

The glomerular filtration rate (GFR) was estimated by the determination of serum creatinine using the equation of the study Modification of Diet in Renal Disease (MDRD) [26]. The diagnosis and stage of the CKD were made according to the criteria of the US National Kidney Foundation [27]. Patients with two results of GFR <60 mL/min/1.73 m^2^, measured at least 3 months apart, were diagnosed with CKD [27]. The outcomes were cardiovascular death or hospitalization secondary to decompensated CHF, during the 12 months of follow up.

The Kolmogorov-Smirnov study was used for evaluation of the presence or not of normal distribution of the variables. The data were expressed as mean and standard deviation and percentages. The population was divided into stages B and C of CHF. To compare the qualitative variables among the two groups, we utilized the chi-squared test or the Fisher's test, and for numerical variables the Student's t test or the Mann-Whitney test.

The univariate analysis was used to evaluate the association between the stages of CHF and the occurrence of outcomes. The multivariate analysis was employed by the backward logistic regression method in order to evaluate the relationship between outcomes, stages of CHF, and the covariates that presented p-valor ≤ 0.1 in the univariate analysis. In the first stage of regression, we utilized variables for which the p-value in the univariate analysis was less that 0.1. The level of significance was set at 5%. The analyses were performed using the SPSS program for Windows 11.0 (SPSS Inc, Chicago, IL).

## Results

There were 87 eligible patients, of which 83 met the inclusion criteria, 33 (39.8%) in stage B of CHF, and 50 (60.2%) in stage C. The mean age of the patients was 62.6 ± 12 years, and 47 were (56.6%) females. Sedentarism and smoking were presented in 57 (68.7%) and 12 (14.5%) patients, respectively. Of the total patients, 79 (95.2%) were hypertensive, and 41 (49.4%) were type 2 diabetics. Stage C was diagnosed in 50 (60.2%) of the patients, representing the major cause of CHF. With the exception of sedentarism, more frequent in patients with CHF stage C compared to stage B (p = 0.02), there were no statistical differences in the remaining demographic parameters between the two groups. (Table 1) The mean BMI was 30.3 ± 7.8 kg/m^2^, and 33 patients (39.8%) were obese. The mean systolic blood pressures were 134.5 ± 20.3 mmHg and the mean PP was 52.2 ± 16.2 mmHg. The mean values of hemoglobin, blood glucose, total cholesterol, LDL-cholesterol, and triglycerides, were not different after stratification of patients in stages B and C of CHF (Table 1).

**Table 1:** Baseline characteristics of patients with CHF, stratified in stages B and C.

Total patients with CHF				
**Variables**	**Total population**	**Stage B**	**Stage C**	**P**

N	83	39.8% (33/83)	60.2% (50/83)	
Age (years)	62.7 ± 12.1	60.4 ± 11.8	64.2 ± 12.2	0.16
Females	(47) 56.6%	(17) 55.4%	(30) 63.8%	0.44
Arterial hypertension	(79) 95.2%	(31) 93.9%	(48) 96%	0.66
Type 2 diabetes mellitus	(41) 49.4%%	(18) 54.5%	(23) 46%	0.44
Sedentarism	(57) 68.7%	(18) 54.5%	(39) 78%	0.02
Smoking	(12) 14.5%	(6) 18.2%	(6) 2%	0.31
Ischemic Cardiopathy	(50) 60.2%	(23) 69.7%	(27) 54%	0.15
BMI	30,3 ± 7,8	28.7 ± 6.2	31.3 ± 8.7	0.12
Obesity	(33) 39,8%	(10) 30.3%	(23) 46%	0.11
SBP	134.5 ± 20.3	131.8 ± 18.6	136.2 ± 21.4	0.33
Hemoglobin (g/%)	13.5 ± 1.4	11.3 ± 0.35	11.1 ± 0.9	0.76
Fasting blood glucose (mg/dL)	128 ± 51.4	124.9 ± 45.6	119.6 ± 55.4	0.65
Total cholesterol (mg/dL)	198.2 ± 49.3	199.7 ± 47.1	197.2 ± 51.2	0.82
LDL-cholesterol(mg/dL)	122.4 ± 38.8	123.9 ± 40.9	121.4 ± 37.9	0.77
Triglycerides (mg/dL)	164.5 ± 99.5	160.3 ± 97.0	167.3 ± 102	0.75
Serum Sodium (mEq/L)	139.4 ± 3.3	138.9 ± 3.5	139.7 ± 3.2	0.26
Echocardiogram: LA	43.1 ± 7.8	42.5 ± 6.1	43.6 ± 8.9	0.50
DDLV	63.5 ± 12.6	61.7 ± 8.2	64.7 ± 14.9	0.30
EF	37.8 ± 7.9	39.0 ± 6.8	37.0 ± 8.5	0.30
Creatinine (mg/dL)	1.3 ± 0.8	1.4 ± 1	1.3 ± 0.6	0.35
GFR	61.7 ± 22.9	61.4 ± 21.5	61.9 ± 24.1	0.90
Chronic kidney disease	(41) 49.4%	(14) 42.4%	(27) 54%	0.05

Where: CHF = Chronic heart failure; BMI = Body mass index (kg/m^2^); SBP = Systolic blood pressure (mmHg); LA = left atrium (mm), DDLV = diastolic diameter of the left ventricle (mm); EF = ejection fraction (%). Data expressed as mean ± Standard deviation or number (n) and percentages (%). GFR = glomerular filtration rate (ml/min/1.73 m^2^).

The measurement of left atrium, left ventricular diastolic diameter, and ejection fraction on the echocardiogram were 43.1 ± 7.8 mm, 63.5 ± 12.6 mm and 37.8 ± 7.9%, respectively, and did not reach statistical differences after CHF staging (Table 1).

The evaluation of the renal function by creatinine and GFR did not differ statistically between the two stages of CHF. CKD (GFR <60 mL/min/1.73 m^2^) was diagnosed in 41 (49.4%) patients, 14 (42.4%) in the stage B of CHF and 27 (54%) in the stage C (p = 0.05) (Table 1). The absolute majority of patients (85.4%) presented GFR between 30-59 ml/min/17.3 m^2 ^(stage 3 of CKD).

The stratification of patients according to occurrence or not of CKD and stages of CHF did not present evidence of differences among demographic characteristics, physical exam, laboratory parameters, or baseline echocardiogram findings, with the exception of an elevated serum creatinine in patients with CHF and CKD (p = 0.02) (Table 2).

**Table 2:** Baseline characteristics of CHF stratified by stages and presence or not of CKD.

	Patients with CKD				Patients without CKD			
**Variables**	**Total**	**Stage B**	**Stage C**	**p**	**Total**	**Stage B**	**Stage C**	**p**

N	41/83	42.4% (14)	54% (27)	0.05	42/83	45.23% (19)	54,76% (23)	0,45
Age (years)	66.9 ± 11.3	65.5 ± 12.4	67.6 ± 10.7	0.57	58.5 ± 11.6	56.6 ± 10	60 ± 12,7	0,34
Females	(23) 56%	(3) 33.3%	(12) 66.7%	0.73	(18) 42,8%	(10) 52,6%	(8) 36,6%	0,24
Arterial hypertension	(40) 97.6%	(14) 100%	(26) 98%	0.46	(39) 97,6%	(17) 89,5%	(22) 95,6%	0,42
DM2	(19) 46.3%	(7) 50%	(12) 44.5%	0.73	(22) 52,4%	(11) 57,9%	(11) 50,3%	0,21
Sedentarism	(31) 75.6%	(9) 64,3%	(22) 81.5%	0.22	(26) 61,9%	(9) 47,4%	(17) 75,8%	0,07
Smoking	(3) 7.3%	0	(20) 7.4%	0.30	(10) 23,8%	(6) 31,6%	(4) 17,4%	0,23
Ischemic cardiopathy	(25) 61%	(11) 78.6%	(14) 51.8%	0.096	(25) 59,5%	(12) 63,2%	(13) 59,3%	0,45
BMI	28.2 ± 6.3	27.5 ± 6.9	29.3 ± 6	0.50	31,8 ± 8,8	29,6 ± 5,6	33,5 ± 10,5	0,15
Obesity	(14) 34.1%	(3) 21.4%	(11) 40.7%	0.21	(19) 45,2%	(7) 38,8%	(12) 53,8%	0,24
SBP	134.1 ± 19.9	135 ± 22.4	133.7 ± 19	0.84	134,8 ± 20,8	129,5 ± 15,4	139,1 ± 23,9	0,14
Hemoglobin (g/%)	13.2 ± 1.6	13 ± 1.9	13.3 ± 1.4	0.62	13,7 ± 1,2	14 ± 1,5	13,4 ± 0,8	0,75
Blood Glucose*	114.4 ± 40.2	115.7 ± 37.7	113.6 ± 42	0.87	128 ± 59,1	131,6 ± 50,5	125 ± 66,4	0,72
Total cholesterol*	200.4 ± 49.5	207 ± 45.5	197 ± 52	0.54	197,9 ± 49,2	194,3 ± 48,7	200,9 ± 50,5	0,67
LDL-cholesterol*	128.7 ± 44.5	132.5 ± 46.7	126.6 ± 44.1	0.69	165,5 ± 40,3	117,3 ± 35,6	118,4 ± 28,3	0,91
Triglycerides*	162.5 ± 94.7	164.7 ± 93.7	161.4 ± 97	0.91	167,5 ± 103	157,1 ± 101,8	176 ± 105,5	0,56
Echocardiogram: LA	42.5 ± 7.7	41.8 ± 6.1	42.8 ± 8.5	0.69	43,8 ± 6,12	43 ± 6,3	43,1 ± 12,6	0,98
DDLV	60.9 ± 7.5	60.5 ± 6.4	61.1 ± 8.1	0.83	66,5 ± 9,3	62,7 ± 9,5	69 ± 19,6	0,23
FE	36.4 ± 8	38.4 ± 6.8	35.4 ± 8.4	0.26	39,2 ± 7,6	39,5 ± 6,9	38,9 ± 8,3	0,79
Creatinine *	1.7 ± 0.1	1.9 ± 1.5	1.5 ± 0.5	0.25	0,9 ± 0,2	0,9 ± 0,2	0,8 ± 0,2	0,02
GFR	42.8 ± 12	41.7 ± 15.1	43.2 ± 10.3	0.71	79,9 ± 14,3	75,9 ± 11,5	83,3 ± 15,7	0,09

Where: CHF = Chronic heart failure; DM2 = type 2 diabetes mellitus; BMI = Body mass index kg/m^2^; SBP = Systolic blood pressure, mmHg; LA = left atrium (mm); DDLV = diastolic diameter of the left ventricle (mm); EF = ejection fraction (%); GFR = glomerular filtration rate, ml/min/1.73 m^2^. * = mg/dL. Data expressed as mean ± standard deviation or number (n) and percentages (%).

**Figure 1:**
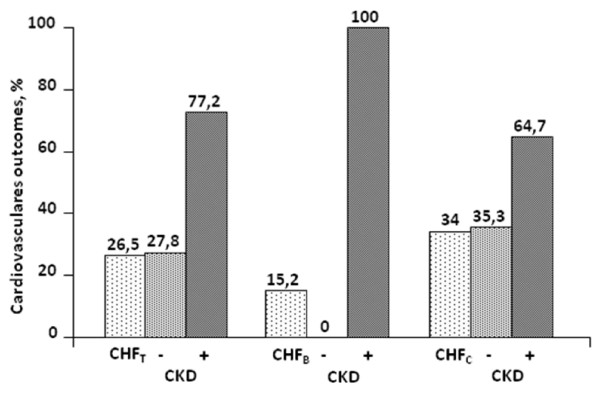
**Presents the cardiovascular outcomes related to the presence or not of chronic kidney disease (CKD) in patients with the chronic heart failure as whole (CHF_T_), and after stratification by stages B (CHF_B_) and C (CHF_C_)**.

The figure 1 presents the cardiovascular outcomes related to the presence or not of CKD in the group as whole, and after stratification by stage. In general, the concurrence of CKD imposes a worse prognosis in patients with CHF. CV outcomes were observed in 22 (26.5%) patients, of which 17 (77.2%) presented CKD. The separation by stages of CHF showed that the presence of CKD was associated with CV outcomes in 5 (100%) patients in the stage B (p = 0.005) and in 8 (64.7%) in the stage C (p = 0.05).

Table 3 presents the univariate and multivariate analyses with Cox's regression model for the clinical prognostic factors determining unfavorable CV outcomes. The univariate analysis showed that sedentarism (RR 3.83; CI95% 1.021-14395; p = 0.04), stage C of CHF (RR 2.885; CI95% 0.944-8.816, p = 0.06), increased LDL-cholesterol (RR 0.354; CI 95% 0.120-1.044; p = 0.06), serum sodium (RR 0.825; CI95% 0.694-0.981; p = 0.02), left ventricular ejection fraction (RR 0.910; CI95% 0.854-0.970; p = 0.004), and CKD (RR 2.891; CI95% 1.052-7.949; p = 0.04) were significantly associated with CV outcomes. The multivariate regression analysis for clinically relevant prognostic factors identified in the univariate analysis showed that the presence of CKD, independently, increased by 3.6 times the chances of occurrence of CV events (CI 95% 1.04-12.67; p = 0.04), while elevated levels of serum sodium (RR 0.807; CI 95% 0.862-0.992; p = 0.03) and ejection fraction (RR 0.925; CI 95% 0.862-0.942; p = 0.03) showed to be protective.

**Table 3:** Univariate and multivariate analysis of the determinants of cardiovascular outcomes in patients with chronic heart failure

	Univariate analysis			Multivariate analysis		
**Variables**	**Risk**	**CI95%**	**p value**	**Risk**	**CI95%**	**p valor**

Chronic kidney disease	2.891	1.052-7.949	0.04	3.641	1.045-12.679	0,042
Stage C of Cardiac heart failure	2.885	0.944-8.816	0.06	2.974	0.785-11.269	0,109
Ejection fraction (%)	0.910	0.854-0.970	0.004	0.925	0.862-0.942	0,03
LDL-cholesterol abnormal	0.354	0.120-1.044	0.06	0.417	0.109-1.593	0,201
Sedentarism	3.830	1.021-1.395	0.04	2.012	0.462-8.775	0,352
Sodium	0.825	0.694-0.981	0.03	0.807	0.862-0.992	0,03

Where: LDL-cholesterol abnormal = serum levels greater than 100 mg/dL.

## Discussion

Our study demonstrated that in the cohort of patients studied, CKD was highly prevalent in stages B and C of CHF, and significantly associated with a greater occurrence of death and hospitalization due to cardiac causes. The unfavorable impact of concomitant occurrence of CKD and CHF was observed in stages B and C of cardiac disease. These findings complement previous observations in patients with more severe forms of CHF (NYHA class IV), that CKD is a frequent complication with an unfavorable impact in the course of the disease [9,11,15,17,19,23,28,29].

Estimation of the GFR used to define glomerular filtration rate (GFR) and CKD stages according to the criteria of the US National Kidney Foundation is considered the new standard of evaluation and severity of renal diseases [12,27]. So far, the utility of this system of staging based on the GFR as a risk factor for prediction of adverse outcome has not been extensively evaluated in patients in less advanced stages of CHF [16,28]. The cohort studied, after adjustment of other important risk factors for unfavorable CV outcomes presented at the baseline period, demonstrated that the occurrence of CKD independently determined a greater occurrence of mortality and hospitalization, an observation still little explored in outpatients with CHF.

The greater occurrence of CV outcomes in patients with CHF and CKD can be explained by the overlap of risk factors common in both diseases separately [30-32]. Previous studies, limited to analyses of databases and stratification of patients into levels of renal function based on dichotomous divisions or tertiles, have demonstrated an association between increased mortality and renal function in patients with CHF [33]. Our findings of CKD as an independent risk factor for CV outcomes is in accordance with other large cohort studies based on communities and other populations with cardiovascular diseases [6,8,12,13,15,16,18,34,35].

The natural course of CHF in its initial stages associated with CKD is still unknown, since, thus far, most studies have evaluated primarily hospitalized patients with severe form of the disease (NYHA class IV), mainly in cross-sectional analyses, and improperly using serum creatinine as a diagnosis of CKD and sometimes excluding patients with CKD from the analysis [36].

In our study, it was observed that CKD (GFR <60 mL/min/1.73 m^2^) present in stages B and C of CHF was associated with 100% and 64.7% of the CV outcomes, respectively. These results provide new information regarding the "natural" course of systolic CHF in its early stages, and if confirmed in studies with greater number of patients, they can define renal function staging as a major tool to identify risk factors for adverse outcomes in patients with less severe CHF.

The unfavorable impact of CKD in the evolution of CHF stages B and C imposes a better understanding of the reasons for such an adverse association. In CHF, low cardiac output, neurohumoral stimulation, aggressive use of diuretics, treatment with renin-angiotensin-aldosterone system blockers, anemia, and comorbidities, such as hypertension and diabetes mellitus, can contribute to the reduction of GFR, and the observed functional deficit merely reflects the severity of CHF in the baseline period [34-37]. However, it is important to highlight that our study only included outpatients already on optimized treatment for CHF. Furthermore, we did not observe statistical differences in the frequency of diabetes and hemoglobin levels in patients with CHF, with and without CKD, which eliminates the possibility of these parameters as determinants of adverse outcomes. These findings reinforce our proposal that CKD *per se*, due to its own risk factors (pro-inflammatory markers, vascular rigidity, dyslipidemia, hyper-homocysteinemia, proteinuria, hypervolemia, and metabolic alterations of calcium and phosphorus), is an important determinant of the observed increase in CV outcomes [37]. In an attempt to better evaluate the unfavorable impact of CKD in the evolution of CHF, we utilized a multivariate analysis to test the hypothesis that renal dysfunction is an independent determinant of poor prognoses, or merely a complication of CHF. We adjusted for other covariables, such as CKD in the baseline period, stage C of CHF, ejection fraction, elevated LDL-cholesterol levels, sedentarism, and serum sodium; and despite these adjustments, the concomitance of CKD increased 3.6 times the occurrence of CV events. This finding reinforces our hypothesis that CKD, independently of the presence of other known risk factors, contributes to the higher occurrence of death and hospitalization for cardiac causes in the early stages of CHF.

It is important to recognize the limitations of our study. Renal function was evaluated by the GRF estimated from creatinine, and not by the use of methods considered to be the gold standard, such as inulin clearance [27]. In addition, we chose to exclusively utilize the functional component of the definition of CKD, in other words, GFR <60 mL/min/1.73 m^2^; however, previous studies have demonstrated that it is below this level of renal function that the risk of mortality increases for all causes of CHF [12,34]. Albuminuria, the main marker of structural renal injury, another component of the definition of CKD, is fundamental in patients with GF >60 ml/min/1, 73 m^2^, and was not determined in this study since its occurrence could be mitigated by the use of optimized angiotensin-converting enzyme inhibitors, AT1 blockers, and aldosterone antagonists, thereby inducing false negative diagnoses [37]. Additionally, our patients were selected from an outpatient clinic attended mainly by hypertensive, diabetic, and obese patients, and thus, the cohort did not entirely reflect the different etiologies of CHF, but, of course, the most prevalent.

### Clinical implications

CKD, a recognizable common risk factor for adverse outcomes in more severe forms of CHF, is also frequent in patients in the early stages of the disease. Our results corroborate with the proposal for using GFR, mainly when it is <60 mL/min/1.73 m^2^, as a strong prognostic risk factor for adverse CV outcomes in CHF, also in patients with less advanced heart failure. These findings suggest that clinical physicians, particularly cardiologists, should incorporate the serial estimation of GFR in order to optimize the results of treatment in CHF. Future studies could elucidate the determinants of the decrease in GFR in CHF stages B and C, and whether or not preservation of renal function will associate with an improved outcome of the cardiac disease.

## Conclusion

In the cohort studied, composed predominantly of hypertensive, diabetic, and obese patients, CKD was common and associated with an increased frequency of cardiovascular outcomes. These findings should be confirmed in studies with larger number of patients and greater diversity of causes of left ventricular dysfunction, in order to better understand the impact of renal function in the course of CHF, and to stimulate the development of new therapeutic strategies, aiming to reduce the rates of hospitalization and mortality, secondary to cardiovascular diseases.

## Conflict of interests

The authors declare that they have no competing interests.

## Authors' contributions

HS have been involved in drafting the manuscript and revising with important intellectual content. AC participated in the design of the study and performed the statistical analysis and contributions to interpretation of data. DM has been involved in drafting the manuscript and revising. MG conceived of the study, and participated in its design, contributions to interpretation of data and coordination. All authors read and approved the final manuscript.

## Pre-publication history

The pre-publication history for this paper can be accessed here:

http://www.biomedcentral.com/1471-2369/10/31/prepub
